# Carnosine, Zinc and Copper: A Menage a Trois in Bone and Cartilage Protection

**DOI:** 10.3390/ijms242216209

**Published:** 2023-11-11

**Authors:** Valeria Ciaffaglione, Enrico Rizzarelli

**Affiliations:** 1 Institute of Crystallography, National Council of Research (CNR), P. Gaifami 18, 95126 Catania, Italy; valeria.ciaffaglione@ic.cnr.it; 2 Department of Chemical Sciences, University of Catania, Viale Andrea Doria 6, 95125 Catania, Italy

**Keywords:** copper, zinc, carnosine, arthritis, bone, cartilage

## Abstract

Dysregulated metal homeostasis is associated with many pathological conditions, including arthritic diseases. Osteoarthritis and rheumatoid arthritis are the two most prevalent disorders that damage the joints and lead to cartilage and bone destruction. Recent studies show that the levels of zinc (Zn) and copper (Cu) are generally altered in the serum of arthritis patients. Therefore, metal dyshomeostasis may reflect the contribution of these trace elements to the disease’s pathogenesis and manifestations, suggesting their potential for prognosis and treatment. Carnosine (Car) also emerged as a biomarker in arthritis and exerts protective and osteogenic effects in arthritic joints. Notably, its zinc(II) complex, polaprezinc, has been recently proposed as a drug-repurposing candidate for bone fracture healing. On these bases, this review article aims to provide an overview of the beneficial roles of Cu and Zn in bone and cartilage health and their potential application in tissue engineering. The effects of Car and polaprezinc in promoting cartilage and bone regeneration are also discussed. We hypothesize that polaprezinc could exchange Zn for Cu, present in the culture media, due to its higher sequestering ability towards Cu. However, future studies should unveil the potential contribution of Cu in the beneficial effects of polaprezinc.

## 1. Introduction

The term “arthritis” usually describes a large family of musculoskeletal architecture disorders; among the different skeletal diseases, osteoarthritis (OA) and rheumatoid arthritis (RA) are the most prevalent [1]. RA is an autoimmune disorder that degrades the joint system, inducing inflammatory insults [2], while OA not only causes cartilage lesions but also gives rise to subchondral bone damage [3]. Trace metals, such as copper (Cu) and zinc (Zn), are essential for skeleton health and regeneration [4]. Bone and cartilage are influenced by Zn^2+^ and Cu^2+^ ions that regulate the tissues’ formation, metabolism, and homeostasis under physiological conditions [5,6,7]. Changes to the normal trace metal levels can alter bone turnover, increasing the risk of fractures. Indeed, these metal ions are not only components of bone and cartilage tissues but also cofactors of enzymes and modulators of osteoblasts, osteoclasts, and chondrocytes’ differentiation and activities [8], as further described in Section 3. Carnosine (Car) is an endogenous dipeptide also involved in bone metabolism with stimulatory activity towards osteoblasts and chondrocytes proliferation and differentiation [9].

Alterations of Cu and Zn homeostasis feature the etiology and pathogenesis of OA and RA [10,11], while Car is suggested as a potential biomarker for skeletal muscle loss, one of the main metabolic alterations occurring in RA and associated with inflammation [12]. Furthermore, Car mitigates OA insults [13] and its conjugate with hyaluronic acid (HyCar) shows protective effects in RA animal models not statistically different from methotrexate [14]. Zinc(II) complex with Car, named polaprezinc, previously employed as an antiulcer and anti-inflammatory drug [15,16], has recently been reported to promote bone fracture healing [17]. This attempt at drug repositioning is inspired by polaprezinc pro-osteogenic effects [18]. In addition, a recent report indicates that Cu interaction with Car and HyCar promotes the nuclear translocation of nuclear factor erythroid 2-related factor (Nrf-2) that regulates the expressions of target genes, including heme oxigenase-1 (HO-1), thus stimulating an antioxidant response in osteoblasts subjected to an inflammatory/oxidative insult [19]. Therefore, these recent studies also highlight the joint activity of the natural peptide and the two biometals, Zn and Cu, to contribute to the skeleton protection against degenerative insults.

Historically, the first studies that inspired the research on the role of trace metals, such as Cu, in rheumatoid joints and inflammation date back to 1939, when Hangarter W. observed that Finnish Cu miners were not affected by arthritis, which was a widespread disease in Finland [20]. Subsequent studies tried to unveil the mechanisms behind trace metals’ protective effects. For instance, Scuderi P. suggested that Cu and Zn may regulate cytokines secretion in 1990 [21]. Moreover, Vinci C. et al. demonstrated that Cu can suppress interleukin 1 (IL-1) activity, explaining the metal chondroprotective effect against synovial-induced damage [22]. Further studies aimed to investigate the role of trace metals in in vitro models of RA inflammation. Of note is that Pasqualicchio M. and colleagues provided evidence of Cu’s ability to protect cartilage against synovial-induced proteoglycan depletion [23].

The first part of this review focuses on emerging studies on Zn and Cu dyshomeostasis in arthritic diseases. Afterward, we describe the unique effects of the trace metals (Zn and Cu), Car, and its Zn complex in bone and cartilage. Finally, we hypothesize that polaprezinc could exchange Zn for Cu due to the presence of Cu^2+^ ions in the culture media and the higher sequestering capacity of Car towards Cu compared to Zn.

## 2. Copper and Zinc Dyshomeostasis in Arthritis

**Figure 1:**
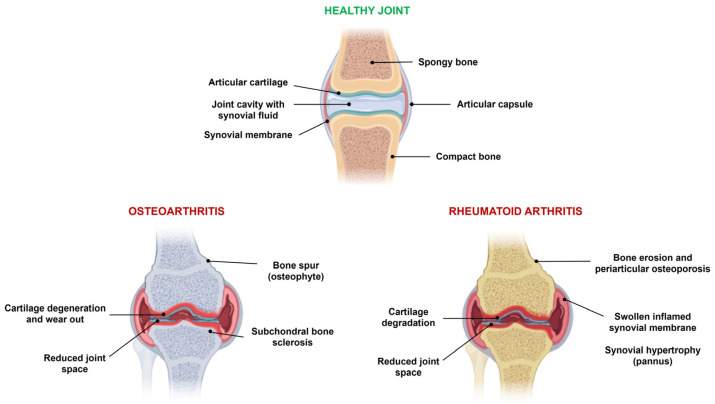
Comparison between healthy joints and OA and RA joints with the typical pathological alterations.

OA is the most prevalent chronic disorder whose clinical symptoms include pain, joint stiffness, swelling, and movement limitations due to the erosion of articular cartilage, hyperplasia and inflammation of the synovial membrane, subchondral bone remodeling, and increased angiogenesis (Figure 1) [24,25].

OA pathogenesis is still poorly understood; however, research on the signaling pathways involved in OA development and progression has increased in the last few years [25]. In this context, inflammatory molecules secreted by the synovial tissue play a substantial role in the pathogenesis of OA [26]. The major proinflammatory cytokines involved are IL-1β and tumor necrosis factor (TNF), produced by osteoblasts, chondrocytes, and synoviocytes. These molecules, independently or jointly with other cytokines and chemokines, trigger the inflammatory cascade and mediate the destruction of the articular cartilage matrix through mechanisms that involve several signaling pathways [27]. Among them, the nuclear factor kappa-light-chain-enhancer of activated B cells (NF-KB) signaling pathway is one of the most important [28]. The result is an altered balance between anabolic and katabolic processes. Moreover, IL-1β and TNF induce the release of catabolic enzymes responsible for cartilage destruction, such as the matrix metalloproteinases MMP-1, MMP-3, and MMP-13 [29], and members of the ADAMTS (a disintegrin-like and metalloproteinase with thrombospondin type 1 motifs) family, such as ADAMTS-4 and ADAMTS-5 [30]. Other signaling cascades are also involved in the onset and development of OA, including wingless-related integration site (Wnt), adenosine monophosphate (AMP)-activated protein kinase (AMPK), mechanistic target of rapamycin kinase pathway (mTOR), hypoxia-inducible factors (HIFs), transforming growth factor-beta (TGF-β) and bone morphogenetic protein (BMP) signaling pathways [25]. Moreover, OA chondrocytes undergo cell death and trigger extracellular matrix (ECM) degradation [31,32,33,34,35].

RA is triggered by the immune system that causes joint inflammation, swelling, pain, cartilage, and bone destruction (Figure 1). If untreated, the disease leads to permanent joint damage, ankylosis, hyperplastic synovium, rheumatoid nodules, vasculitis, progressive deformities, disability, and premature death. Several factors may contribute to the development of RA. In addition to genetic predisposition, respiratory exposure to noxious agents, intestinal health, lifestyle, and habits affect the occurrence of the disease [36]. The interactions between non-immune cells of the synovial tissue, fibroblast-like synoviocytes (FLSs), with monocytes, macrophages, T lymphocytes, and B cells play a key role in RA pathogenesis [37]. The resulting cascade of reactions leads to the production of local autoantibodies, including rheumatoid factor and anti-citrullinated peptide antibodies, and pro-inflammatory factors [38]. Moreover, FLSs acquire an aggressive phenotype in RA and act as direct effectors of joint degeneration through the production of cytokines, chemokines, and pro-angiogenic mediators that sustain the inflammatory state and the secretion of proteases, like MMPs, that degrade the ECM [39].

Oxidative stress is a common factor implicated in the pathogenesis of OA and RA. Indeed, oxidative stress in arthritic patients results from disrupted endogenous antioxidant defense systems and consequent increased pool of reactive oxygen species (ROS) and reactive nitrogen species (RNS) in the inflamed joints [40] and cartilage [41]. Moreover, ROS can activate the NF-KB signaling pathway, thus sustaining the inflammatory state [42]. However, more in-depth studies are required to understand the exact role of oxidative stress in RA and OA initiation and maintenance. In addition, there is evidence of the involvement of metal dyshomeostasis in the pathogenesis of arthritis disorders [43,44]. In particular, imbalances in the levels of Zn and Cu characterize the serum samples of arthritis patients and seem to be related to the inflammatory state and disease activity [45].

Zn and Cu can influence arthritic disease onset and progression since they play a pivotal role in bone and cartilage metabolism, innate and adaptive immunity, inflammation, and antioxidant defense [10,46,47]. To date, numerous studies have supported an altered metal homeostasis in the serum of RA patients [11]. Meta-analyses were performed to overcome conflicting data reported in the literature and better estimate the changes in Cu/Zn ratio between the serum of RA patients and healthy subjects. A meta-analysis by Xin L. et al. conducted in 2015 took into account 26 studies and a total of 1444 patients with RA and 1241 healthy controls [11]. The overall results showed that the mean levels of Cu in the serum of RA patients were higher compared to the healthy controls, while the levels of Zn were lower. Changes in the Cu/Zn ratio in blood samples of RA patients were also investigated in a more recent meta-analysis published in 2019 [43]. In agreement with the previous one, 27 and 23 studies examined serum Cu and Zn levels, respectively, highlighting an increase in Cu and a decrease in Zn and selenium (Se) concentrations and suggesting that trace metal ions may take part in RA pathogenesis. The same study also showed that Cu levels are directly proportional to the duration of the disease. Moreover, subgroup analysis revealed that patients have diverse serum Cu and Zn levels across countries, probably due to different ethnicities, soil environments, and dietary habits [43]. Wang H. et al. performed a more recent study, published in 2023, which further supports the evidence of a higher Cu/Zn ratio in the blood samples of RA patients compared to those of controls [45]. This study investigated the link between Cu levels, inflammation, and various parameters of disease activity. Of note is that a positive correlation was found between serum Cu concentration, inflammatory markers, such as C-reactive protein (CRP), and erythrocyte sedimentation rate, and the disease activity, according to the Disease Activity Score 28 with CRP (DAS28-CRP), that evaluate the severity of RA. However, no correlation was found between serum Zn content and the risk of high RA activity. The same study also took into account iron (Fe) blood levels, which were lower in RA patients compared to healthy controls. Another recent study confirmed that the Cu/Zn ratio could be useful in the diagnosis and prognosis of RA, as well as a marker of altered antioxidant defense systems in arthritic patients [48]. The authors found a decrease in concentration of the antioxidant tripeptide glutathione (GSH) and an increase in malondialdehyde (MDA), a lipid peroxidation marker in RA patients, further supporting the involvement of oxidative stress in the pathogenesis of the disease.

**Figure 2:**
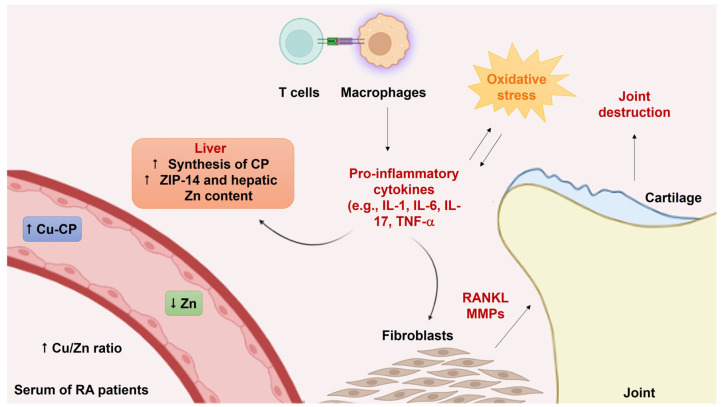
The putative linkage between inflammatory cytokines and increased Cu/Zn ratio in the serum of RA patients. Activation of T cells and macrophages leads to pro-inflammatory cytokines production, such as IL-1, IL-6, IL-17, and TNF-α. Cytokines induce fibroblasts to act as effectors of joint destruction through the release of other cytokines, chemokines, and proteases, like MMPs. Moreover, the pro-inflammatory cytokines stimulate the hepatic synthesis of CP, with consequent increased levels of Cu-CP in the blood. In addition, pro-inflammatory cytokines cause the overexpression of the Zn importer ZIP-14 in the liver. These effects lead to an increased Cu/Zn ratio in the serum of RA patients. The overall inflammatory state enhances ROS generation and oxidative stress, which in turn sustains inflammation and tissue injury. The symbols “↑” and “↓” stand for increase and reduction, respectively.

The discussed meta-analyses suggest that the altered status of trace elements in RA may be either a causative factor in the disease’s pathogenesis or a consequence of the inflammatory condition [49]. Cu amount is tightly regulated because metal overload can catalyze ROS production through Fenton-like reactions, causing oxidative damage [50]. Cellular Cu homeostasis is controlled by a complex network of proteins and chaperones that started to be elucidated in the early 1990s [51,52,53]. In this context, a key role is exerted by the Cu transporters family CTR, encoded by the solute carrier gene *SLC31*. In particular, this family of proteins includes CTR1 and CTR2, encoded by *SLC31A1* and *SLC31A2* genes, respectively, which act as influx transporters [54]. CTR1 is mainly located on the cell membrane and shows a high affinity for Cu, whereas CTR2 is largely associated with intracellular vesicles and shows a lower affinity for Cu [55]. CTR1 and CTR2 are ubiquitously expressed, mediating Cu uptake in all organs and tissues. The average concentration of Cu in human blood plasma is in the range of 12.7–22.2 μM under physiological conditions [56,57]. The vast majority of Cu in plasma is bound to ceruloplasmin (CP) (approximately 75%) and other Cu-binding proteins, such as albumin [58]. CP is a serum ferroxidase mainly produced in the liver, where Cu^2+^ ions are incorporated into apo-CP, serving as catalytic centers. Although the mechanisms responsible for an increase in the Cu/Zn ratio have not been fully elucidated, a relationship between inflammatory mediators and the serum concentration of these trace metals has been reported [59]. Figure 2 shows the putative correlation between increased Cu/Zn ratio in the serum of RA patients and inflammation. Indeed, inflammatory cytokines, including interleukin IL-6, IL-1β, TNF-α, and interferon-γ (IFN-γ), can increase the hepatic synthesis of CP and its secretion into the blood [60]. A recent study further explored the link between serum Cu and the progression of RA [61]. Interestingly, Aldabbagh K.A.O. and co-workers reported that high Cu levels are positively correlated to the activity and severity of the disease, confirming the potential diagnostic and prognostic value of serum Cu concentration in RA management.

Zn is regulated at tissue, cellular, and subcellular levels by sophisticated systems that mediate its transport, storage, and distribution. In particular, more than 30 transport proteins are involved in Zn efflux and export, which are mainly represented by two different families of Zn transporters, the Zn transporter (ZnT) and Zrt- and Irt-like protein (ZIP) families, encoded by the *SLC30* and *SLC39* genes, respectively [62]. Inflammatory mediators may contribute to reducing serum Zn levels. Indeed, IL-6 can upregulate the Zn importer ZIP-14 in the liver, thus reducing Zn amounts in the serum and increasing the hepatic Zn content (Figure 2) [63,64]. In addition, in vivo experiments suggested that oral Zn supplementation could be beneficial in RA patients. A double-blind trial showed that zinc sulfate administration for 12 weeks could improve the patients’ clinical parameters, such as joint swelling, morning stiffness, and overall articular function [65].

Unfortunately, poor data have been reported on altered blood levels of Zn and Cu in OA so far [66]. Unlike RA, changes in Cu concentrations in synovial fluid and plasma have not been found in OA patients [67]. However, a recent study suggested that a genetic predisposition to high Cu and Zn circulating levels is associated with OA [68].

In conclusion, the results of the aforementioned meta-analysis are in agreement and suggest that the increased Cu/Zn ratio could be associated with inflammation, oxidative stress, and RA pathogenesis. However, whether metal dyshomestasis in the serum of arthritic patients is simply the result of the inflammatory processes or is associated with altered metal concentrations in tissues is still unknown. Zn and Cu detection in different tissues remains a major challenge. Therefore, the potential of these trace metals as biomarkers and their exact role in RA are worthy of further investigation.

## 3. The Bioinorganic Chemistry of Zinc and Copper in Bone and Cartilage Tissues

**Figure 3:**
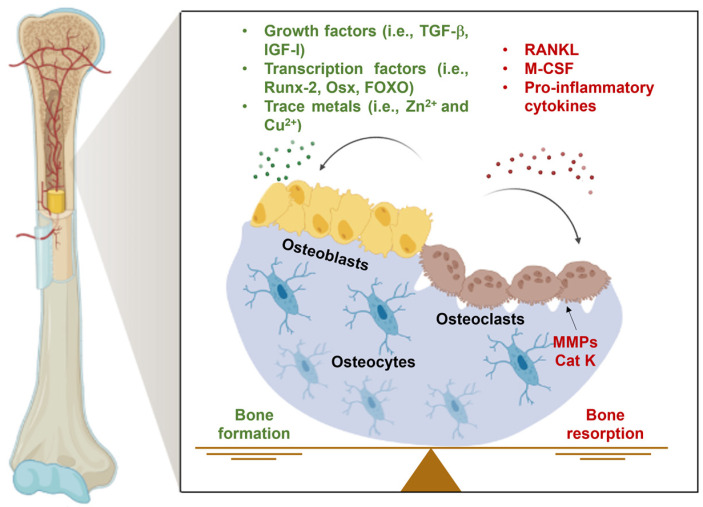
Simplified representation of the dynamic equilibrium between bone formation and resorption and the main signaling molecules involved.

Bone is a dynamic and metabolically active tissue that undergoes continuous remodeling to degrade old or damaged bone and replace it with newly synthesized bone tissue [69]. The bone matrix is composed of a mineral part of hydroxyapatite (approximately 65%) and an organic portion (about 35%), mainly made of matrix proteins, proteoglycans, and collagen. Bone homeostasis is necessary to preserve its functions; the most important ones are (i) providing mechanical support for the soft tissues; (ii) protection of vital organs; (iii) muscle attachment; and (iv) storage for minerals, mainly calcium and phosphate. Bone remodeling requires the coordinated actions of three main types of cells: osteoblasts, osteoclasts, and osteocytes [70]. Osteoblasts are specialized cells that form new bone tissue, while osteoclasts are responsible for bone resorption. The coordinated activities of these cell types regulate the skeleton development and remodeling. Different signaling pathways and molecules have crucial roles in preserving bone health (Figure 3). Among them, there are trophic factors, like BMPs, that belong to the family of TGF-β proteins. Members of the BMPs family, including BMP-2, BMP-4, BMP-6, and BMP-7, control the proliferation and activity of osteoblasts and chondrocytes, inducing the formation of bone and cartilage tissues [71]. Moreover, the signaling systems involved in bone metabolism are regulated by specific transcription factors. Runt-related transcription factor 2 (Runx2), the osteoblast-specific transcription factor osterix (Osx), the forkhead box O (FOXO) family, and T-cell factor/lymphoid enhancer factor (TCF/LEF) are some of the most important ones in promoting bone formation [72,73,74,75]. Trace elements, such as Cu^2+^ and Zn^2+^ ions, also have a crucial impact on bone homeostasis by affecting its metabolism, density, and strength [8]. On the other hand, the resorption of bone by osteoclasts is stimulated by the receptor activator of nuclear factor kappa-light-chain-enhancer of activated B cells (NF-KB) ligand (RANKL), which induces osteoclast activation and maturation. Other factors include macrophage colony-stimulating factor (M-CSF), which secrete catalytic enzymes like MMPs and cathepsin K (Cat K), and cytokines, such as TNF-α, IL-1, IL-6, IL-7, IL-8, IL-11, IL-15, IL-17, IL-23, and IL-34, that induce osteoclasts differentiation [76]. The dynamic equilibrium between osteoblasts and osteoclasts’ activities is controlled by the mechanosensitive bone cells osteocytes, which release signaling molecules, such as osteoprotegerin (OPG), prostaglandin E2 (PGE2), cyclooxygenase-2 (COX-2), and nitric oxide (NO) [77]. Bone production, resorption, and preservation are also subjected to hormonal regulation. Hormones that influence bone remodeling include parathyroid hormone (PTH), calcitonin, growth hormone, and cortisol [78]. Disruption of bone homeostasis and imbalance between osteoblasts’ activity, responsible for new bone formation, and bone resorption by osteoclasts, results in bone mass abnormalities, typical of diseases such as osteoporosis, OA, and bone tumors [79].

Unlike bone, joint cartilage has a poor capacity for self-repair. Indeed, it is an avascular connective tissue adjacent to the subchondral bone, inaccessible to systemic regulation [80]. The main active components of articular cartilage are chondrocytes, which secrete and control the homeostasis of a dense ECM. The latter is rich in water, collagen (mainly collagen type II), proteoglycans, and non-collagenous proteins. Its main function is to allow smooth joint movement with minimal friction and mediate the transmission of loads to the subchondral bone, thus protecting the joints [81,82]. Chondrogenesis is a highly controlled process that leads to skeletal bone development and is mediated by hormones, growth factors, transcription factors, and matrix proteins. The most important transcription factors in cartilage development include SRY-box transcription factor(SOX9/SOX5/SOX6), Runx2/Runx3, and Osx, which regulate the expression of chondrogenic genes, such as *collagen type II alpha 1 chain (Col2a1)*, *collagen type XI alpha 2 chain (Col11a2)*, *collagen type X alpha 1 chain (Col10a1)*, *aggrecan (ACAN)*, *PTH receptor*, *alkaline phosphatase (ALPL)*, and *vascular endothelial growth factor A (VEGF-A)* [83,84]. Given the lack of cartilage’s self-repairing capability, current treatment options, such as subchondral bone drilling, cartilage transplantation, chondrocyte and mesenchymal cell transplantation, help to repair chondral lesions and alleviate the pain [85].

Articular cartilage lesions are often accompanied by damage to the subarticular bone, leading to total joint destruction. However, the development of effective strategies for repairing damaged joints is challenging due to the different natures of bone and cartilage. In recent years, tissue engineering approaches based on the use of stem cells, scaffolds, and growth factors have demonstrated the potential as therapeutic strategies for repairing and restoring osteochondral defects, overcoming the limits of bone diseases’ conventional therapies, such as the lack of supply of autologous bone, immune rejection, and high medical costs [86,87,88]. Several strategies involving the implantation of biomimetic scaffolds into bone or cartilage defects have been explored. Different materials, such as polymers [89], bioceramics [90], hydrogels [91], and alloys [92], were investigated to create optimal scaffolds with biocompatible and biodegradable properties. Many studies focused on developing metal-based biomaterials, thanks to metal ions’ osteogenic and osteo-inductive properties [93,94,95,96]. Metal ions, such as calcium (Ca), magnesium (Mg), phosphorus (P), fluoride (F), Cu, Zn, and Fe, are essential components of various tissues, including bone and cartilage, where they are involved in mineralization, bone formation, and healing processes [97,98].

### 3.1. Zinc

Zn is an essential trace element that takes part in numerous physiological processes of the human body [99]. Approximately 80% of the whole body’s Zn content is localized in muscles and bone, where Zn plays a crucial role in promoting skeleton growth and homeostasis, even though the cellular and molecular pathways involved have not been fully understood yet [100]. It is a structural and catalytical constituent of diverse enzymes and an intracellular and extracellular signaling mediator [101]. In particular, Zn is the catalytic component of more than 300 enzymes and is the only metal found in all six classes of enzymes (hydrolases, lyases, ligases, oxidoreductases, transferases, and isomerases) [102]. As a constituent of metalloproteins, Zn is involved in many physiological functions, such as cell proliferation, nucleic acid synthesis, protein folding, and redox regulation [103]. It generates the “zinc fingers”, small motifs consisting of a Zn^2+^ ion that coordinates cysteine and histidine residues [104]. Zn also shows antioxidant effects since it is necessary for antioxidant enzyme activation, including catalase and proteins like GSH, an essential antioxidant in mammalian cells. [105]. Moreover, it directly binds to thiol groups of proteins, protecting them against irreversible oxidation [106]. Furthermore, Zn participates in anti-inflammatory and immune responses. Indeed, several studies demonstrated that Zn can suppress NF-KB (a pivotal mediator of inflammation) [107] and increase the expression of anti-inflammatory and antiapoptotic proteins [108]. In addition to its catalytic and structural roles, this metal ion is also involved in cellular signaling and transfer of information, especially in the immune and central nervous systems [109].

Zn deficiency is associated with impaired skeletal development in humans and animals [110]. Early studies on the effects of Zn^2+^ ions on bone metabolism were performed more than 60 years ago [111]. In 1961, Prasad and colleagues suggested that dwarfism and hypogonadism observed in short-stature patients could be associated with Zn deficiency [112]. This hypothesis was subsequently confirmed in 1967 by Sandstead H. and coworkers, who showed that dietary Zn supplementation improved the growth of these patients [113]. Following studies reported that altered Zn homeostasis is often related to the onset and progression of diseases and pathological states, including bone growth retardation, aging, and postmenopausal conditions, suggesting that Zn is necessary for maintaining bone homeostasis and promoting bone regeneration [110]. For example, a recent study showed that oral Zn administration can prevent bone destruction and attenuate diabetic osteoporosis, a complication of diabetes, in rats [114]. Moreover, increasing data have shown that dysfunctions of Zn^2+^ transporters, which regulate the influx and efflux of this metal ion, are associated with pathological human bone conditions, as recently reviewed by Huang T. et al. [115].

#### Effects of Zinc in Bone and Cartilage Tissues

Data from the literature demonstrated that Zn contributes to bone mineralization, metabolism, and turnover; therefore, its use in bone tissue engineering has attracted much attention in the last few years [116]. Table 1 summarizes the main effects exerted by Zn-based compounds in bone and cartilage tissues. Firstly, Zn plays an essential role in bone metabolism as the cofactor of enzymes involved in bone matrix mineralization, such as collagenase and alkaline phosphatase (ALP) [117]. The latter is a metalloenzyme highly expressed in the bone that catalyzes the hydrolysis of phosphate esters and facilitates mineralization by releasing phosphate ions in the bone matrix [118]. In vitro experiments on osteoblastic MC3T3-E1 cells suggested that Zn increases ALP synthesis and secretion in a dose-dependent manner and induces collagen synthesis [119]. Zn also showed anabolic effects in vivo through the upregulation of osteocalcin (OCN) and serum ALP activity, and protective effects against diabetes-induced bone loss [120]. Furthermore, Zn^2+^ ions significantly increased the concentration of bone protein components and growth factors, including insulin-like growth factor-1 (IGF-1) and TGF-β, in bone tissues with fracture healing [121,122,123], probably through the signaling pathway of protein kinase C and protein phosphatase [124]. In addition to the modulation of bone metabolism, Zn shows stimulatory effects on bone formation. Indeed, Zn positively modulates osteoblast functions while inhibiting osteoclasts’ activity. Seo et al. showed that treatment with zinc chloride stimulates the proliferation of osteoblastic cells MC3T3-E1 in a dose-dependent manner [119]. In addition to its stimulatory effects on ALP activity and collagen synthesis, Zn treatment can affect osteoblast differentiation by increasing the expression of some of the major differentiation markers, such as Runx2, OPG, and regucalcin, as shown in MC3T3-E1 cells [125]. However, the mechanism involved in Runx2 induction by Zn is still poorly understood. According to Cho E. and coworkers, Zn can induce the expression of osteoblastic differentiation transcription factors, such as Runx2 and Osx, by modulating BMP-2 signaling [126]. Runx2 and Osx, in turn, regulate the transcription of ZIP1 transporter and the consequent influx of Zn^2+^ ions, establishing a Zn^2+^-Runx2/Osx-ZIP1 axis that promotes not only citrate deposition in bone apatite, facilitating bone mineralization, but also osteogenic differentiation of mesenchymal stem/stromal cells (MSCs) [127]. In another study, Park K.H. et al. suggested that Zn can induce Runx2 expression via activation of a cAMP-mediated pathway [128]. Zn ability to promote osteogenic differentiation was also evaluated on adipose tissue-derived mesenchymal stem cells (ADSCs) under an electromagnetic field as a strategy in osteoporosis therapy [129]. Of note is that zinc sulfate in the presence of an electromagnetic field induces osteogenic gene expression, ALP activity, and calcium content. In particular, Zn enhances ADSCs osteogenic differentiation via protein kinase A (PKA), extracellular signal-regulated kinase 1/2 (ERK1/2), and Wnt/β-catenin signaling pathways, as demonstrated by real-time PCR analysis [129]. In addition, Zn shows protective effects towards osteoblasts under oxidative damage [130]. On the other hand, Zn can act as an inhibitor of bone resorption, as shown in in vitro models of osteoclastogenesis [131]. Several studies reported the Zn capability of suppressing osteoclastic bone resorption by direct and indirect mechanisms [132]. For instance, Zn can directly inhibit osteoclastogenesis by suppressing NF-KB signaling, a pathway necessary for osteoclastogenesis, in osteoclast precursors [133]. Other studies demonstrated that Zn^2+^ ions can inhibit osteoclastogenesis by interfering with the Ca^2+^–calcineurin–nuclear factor-activated T cells c1 (NFATc1) signaling pathway [134]. NFATc1 is a transcription factor that modulates osteoclast differentiation [135]. In particular, NFATc1 translocates to the nucleus and induces the expression of various osteoclast-specific genes after calcineurin-induced dephosphorylation. In this context, Zn treatment decreases calcineurin phosphatase activity, thus suppressing RANKL-induced Nfatc1 translocation and inhibiting osteoclast formation from mouse bone marrow-derived monocyte cells (mBMMs) in a dose-dependent manner [134]. Moreover, other studies demonstrated that Zn protective effects against bone loss also occur via RANKL/RANK downregulation [136]. The RANKL/RANK axis plays an essential role in osteoclastogenesis and bone remodeling. For instance, an in vivo study by Ferreira E. and colleagues showed that Zn supplementation preserves bone architecture by reducing the RANKL/OPG ratio in ovariectomized and type 1 diabetic rats [137]. In addition, Zn is able to stimulate osteoclast apoptosis. As shown by Li X. et al., tricalcium phosphate discs containing 0.3 and 6.8 ppm Zn^2+^ increase osteoclast apoptosis of approximately 5% and 30%, respectively, in the culture media after 24 h [138].

Zn^2+^ ions incorporation into biomaterials has been recently studied for bone defects treatment due to Zn osteogenic properties and its ability to modulate bone microenvironment. Wang B. et al. investigated the use of carbon dots doped with Zn^2+^ ions for promoting the adhesion and proliferation of bone marrow stromal cells (BMSCs) and bone formation in a rat model of bone defect [139]. In another recent study, Zn was incorporated into a polycaprolactone (PCL)/gelatin nanofiber through the electrospinning technique, generating an ECM-mimicking support [140]. The potential of Zn^2+^ ions in bone tissue engineering is also related to its antibacterial activity. For example, Zn-implanted titanium stimulates adhesion and proliferation of MC3T3-E1 cells, ALP activity, bone matrix mineralization, and antibacterial activity towards *Escherichia coli* and *Staphylococcus aureus* [141]. The antibacterial effects of Zn were also highlighted in the following studies, such as by Maleki-Ghaleh H. et al., who demonstrated the antimicrobic behavior of Zn^2+^ ions incorporated into hydroxyapatite/graphene nanocomposites [142]. In addition, Zn can create a favorable microenvironment for bone repair thanks to its ability to induce angiogenesis-related gene expression, *VEGF-A* and *cluster of differentiation 31* (*Cd31*) [143]. Finally, Zn can affect chondrogenesis. Altered Zn levels are associated with pathological changes in cartilage tissues. For instance, early studies showed that Zn deficiency significantly suppresses chondrocyte proliferation [144], while Zn treatment enhances the proliferation rates of cultured chondrocytes [145]. Recently, Hozan S. et al. proved that zinc chloride induces the expression of both pro-angiogenic (*VEGF-A* and *VEGF-B*) and pro-chondrogenic (*SOX9*, *Runx1*, *collagen*) genes, suggesting that this metal ion can stimulate chondrogenesis, accelerating the fracture healing process [146]. Another study by Huang T. et al. showed that Zn supplementation prevents cartilage degradation and inhibits monosodium iodoacetate (MIA)-induced OA progress both in vitro and in vivo [147]. Zn protective effects on chondrocytes are due to its anti-inflammatory and antioxidant activity. Indeed, Zn reduces pro-inflammatory cytokines and activates the phosphoinositide 3-kinase (PI3K)/protein kinase B (Akt)/Nrf-2 pathway [147]. The potential application of Zn in repairing bone defects was also investigated in a clinical study [148]. The latter suggested that Zn-incorporated nanohydroxyapatite bone graft is a promising bone regenerative material in chronic periodontitis patients. Therefore, these results demonstrated that Zn non-toxic nature, osteogenic, chondrogenic, angiogenic, and antibacterial properties are desirable features for orthopedic applications.

**Table 1:** Summary of the principal effects exerted by Zn-based compounds/materials in bone and cartilage tissues.

Zn Source	Cell Type	Animal Model	Effects	Outcomes	Ref.
Zinc chloride	MC3T3-E1	-	↑ cell proliferation, ALP activity, collagen synthesis	↑ bone formation	[119]
Zinc carbonate	-	diabetes-induced bone loss in rats	↑ OCN expression, ALP activity	↓ bone loss	[120]
Zinc acexamate	-	rats with fracture healing	↑ IGF-1 and TGF-β	↑ fracture healing	[121]
Zinc sulfate	MC3T3-E1	-	↑ *Runx2*, *OPG*, *regucalcin* mRNA	↑ osteoblast differentiation	[125]
Zinc chloride	MC3T3-E1	-	↑ *Runx2* expression via BMP-2 signaling	↑ osteoblast differentiation	[126]
Zinc sulfate	ADSCs	-	↑ *β-catenin*, *Wnt1*, *Wnt3a*, *LRP5* and *DKK1* mRNA ↑ ALP and PKA activity ↑ Ca^2+^ levels	↑ osteoblast differentiation	[129]
Zinc sulfate	RAW264.7	-	↓ NF-KB activation	↓ osteoclastogenesis	[133]
Zinc sulfate	mBMMs, RAW264.7	-	↓ NFATc1 signaling pathway	↓ osteoclastogenesis	[134]
Zinc sulfate	-	ovariectomized and type 1 diabetic rats	↓ RANKL/OPG ratio	↓ bone alterations	[137]
Zn-containing tricalcium phosphate	osteoclasts isolated from rabbits	-	↓ actin ring formation	↑ osteoclast apoptosis	[138]
Zn-carbon dots	BMSCs	calvarial bone defect model in rats	↑ ALP activity, area of calcified nodules	↑ bone regeneration	[139]
Zn-PCL/gelatin nanofiber	MG-63	-	↑ ALP activity, Ca^2+^ content, ↑ *Runx2* and *COL1* expression	↑ bone regeneration	[140]
Zn-implanted titanium	MC3T3-E1	-	↑ ALP activity, collagen secretion, mineralization, antibacterial activity towards *E. coli* and *S. aureus*	osteogenic, antibacterial effects	[141]
Zn-doped hydroxyapatite/ graphene nanocomposite	MSCs	-	↑ cell proliferation, ALP activity, antibacterial function against *E. coli* and *S. aureus*	osteogenic, antibacterial effects	[142]
Zn silicate/nanohydroxyapatite/ collagen scaffolds	BMSCs	calvarial defect model in rats	↑ *BMP-2*, *Osx*, *VEGF-A*, *Cd31* expression	↑ osteogenesis, angiogenesis	[143]
Zinc chloride	ATDC5	-	↑ *VEGF-A*, *VEGF-B*, *SOX9*, *Runx1*, *collagen* expression	↑ chondrogenesis	[146]
Zinc sulfate	SW1353	MIA-induced OA in rats	↓ pro-inflammatory cytokines ↑ PI3K/Akt/Nrf-2 pathway	protective effects	[147]

Abbreviations: ADSCs—adipose tissue-derived mesenchymal stem cells; Akt—protein kinase B; ALP—alkaline phosphatase; BMP-2—bone morphogenetic protein 2; BMSCs—bone marrow stromal cells; Cd31—cluster of differentiation 31; COL1—collagen type 1; DKK1—dickkopf Wnt signaling pathway inhibitor 1; IGF-1—insulin-like growth factor-1; LRP5—low-density lipoprotein receptor-related protein 5; mBMMs—mouse bone marrow-derived monocyte cells; MIA—monosodium iodoacetate; MSCs—mesenchymal stem/stromal cells; NFATc1—Ca^2+^–calcineurin–nuclear factor-activated T cells c1; NF-KB—nuclear factor kappa-light-chain-enhancer of activated B cells; Nrf-2—nuclear factor erythroid 2-related factor; OA—osteoarthritis; OCN—osteocalcin; OPG—osteoprotegerin; Osx—osteoblast-specific transcription factor osterix; PI3K—phosphoinositide 3-kinase; PKA—protein kinase A; RANKL—receptor activator of NF-KB ligand; Runx1—runt-related transcription factor 1; Runx2—runt-related transcription factor 2; SOX9—SRY-box transcription factor 9; TGF-β—transforming growth factor-beta; VEGF-A—vascular endothelial growth factor A; VEGF-B—vascular endothelial growth factor B; Wnt1—proto-oncogene int-1 homolog; Wnt3a—Wnt family member 3a. The symbols “↑” and “↓” stand for increase and reduction, respectively.

### 3.2. Copper

Cu is the third most abundant essential trace element in the human body involved in a wide range of physiological processes [8]. It is the catalytic center of numerous intracellular enzymes and its main function is to participate in oxidation-reduction processes, acting as either an electron acceptor or donor thanks to its two oxidation states: reduced, Cu^+^, found predominantly within the cell and subcellular compartments, and oxidized, Cu^2+^, present in extracellular tissues and fluids. Diverse metabolic functions, such as energy production, biosynthesis of neurotransmitters, neuropeptide activation, immune function, angiogenesis, and formation of connective tissues, are influenced by Cu [149,150]. Indeed, it is the cofactor of enzymes, such as lysyl oxidase (LOX), responsible for collagen cross-linking in connective tissues [151], superoxide dismutase (SOD), an antioxidant enzyme that catalyzes the dismutation of the toxic superoxide anion radical (O_2_^●−^) [152], and cytochrome c oxidase (Cox), involved in the mitochondrial respiratory chain [153]. Cu^2+^ ions also regulate bone metabolism and accelerate the rate of wound healing, showing similar effects to those observed for Zn^2+^ [5]. In addition, Cu is a proangiogenic and antibacterial agent [154]. Cu deficiency is a rare condition linked to several diseases, such as anemia, neutropenia, and bone alterations due to altered osteogenesis [155]. The vast majority of data on the effects of Cu deficiency on bone is related to studies performed more than 40 years ago on animal models. According to these findings, dietary Cu deficiency causes connective tissue and skeletal defects that could lead to osteoporosis [156,157]. On the other hand, excessive Cu amount can cause ROS production, lipid peroxidation, and inflammation, which are detrimental to bones [158]. However, there is no consensus on the threshold for Cu cytotoxicity. Therefore, a narrow dose range of Cu is necessary to guarantee the physiological processes involved in bone metabolism and remodeling [159,160]. The beneficial properties of Cu in repairing bone, cartilage, and blood vessels and its antibacterial activity made this metal ion a potential candidate for biomaterials development. Various forms of Cu-based biomaterials were developed in recent years, which showed potential applications for bone and cartilage tissue engineering [161,162,163,164]. Of note is that Cu^2+^ ions incorporation into biomaterials has provided promising biological effects, together with improved porosity, mechanical strength, and crosslinking of the scaffolds [165].

#### Effects of Copper in Bone and Cartilage Tissues

The effects of Cu in osteoarticular processes were investigated in in vitro and in vivo assays [166]. Like Zn, Cu is involved in bone mineralization and metabolism, and its deficiency causes bone alterations. Indeed, Cu deficiency can impair monoamine oxidase and LOX activities, reducing bone collagen stability and bone structure’s strength [167]. Several studies provided evidence that Cu can induce biological responses in osteoblast precursors and highlighted its osteogenic properties, for developing Cu-based biomaterials for bone defects repair [168]. Of note is that Burghardt I. et al. reported that Cu release from titanium implants induces the proliferation and osteogenic differentiation of MSCs, increases ALP activity, the expression of collagen I, OPG, osteopontin (OPN), and finally bone mineralization [169], which are the same effects exerted by Zn, previously described. The authors also shed light on the dual function of Cu as both an anti-microbial and regenerative agent, which makes this metal ion particularly promising for the fabrication of orthopedic implants [169]. An important aspect that emerged is that Cu^2+^ ions can achieve a dual activity by tuning the concentration that reaches the target tissue. In particular, higher concentrations of Cu (0.3–1.75 mM), immediately released after implantation, were responsible for an antibacterial activity against *Staphylococcus aureus*. On the contrary, Cu reached more distant sites at lower concentrations (about 0.1 mM), stimulating the proliferation and osteogenic differentiation of MSCs [169]. Cu showed cytotoxic effects on MSCs at concentrations above 0.5 mM. Therefore, it could be possible to use lower concentrations (0.3 mM), which can reduce the growth of the bacteria without harming MSCs. These findings suggested that Cu effects depend on the tested concentrations and experimental conditions and explain the contradictory results on Cu activity (reported as inhibitory or stimulatory) towards osteoblastic cell proliferation [169]. Several studies ascribe its antimicrobial activity to the increased Cu^2+^ ions uptake into bacteria, where Cu causes oxidative stress with consequent damage of cytoplasmic membrane and DNA, and suggest its use in implant materials to prevent infections [170,171]. In addition, the osteogenic activity of Cu has been associated with the regulation of the Wnt/β-catenin signaling [172]. This pathway stimulates the osteogenic differentiation of BMSCs and plays an essential role in controlling bone remodeling [173]. However, alterations in this signaling pathway have been linked to several pathological conditions, including defects in bone mass [174]. Therefore, Wnt/β-catenin signaling has been proposed as a potential therapeutic target for the treatment of osteoporosis [175]. As shown by Tan et al., Cu^2+^ ions released by titanium alloy implants upregulated genes and proteins involved in the Wnt signaling, including axin2, β-catenin, glycogen synthase kinase 3 (GSK-3β), LEF1 and TCF1/TCF7, and promoted osteoporotic fracture healing in vivo [172]. However, excessive Cu concentration exerted an inhibitory effect on the Wnt pathway in fish embryos, suggesting that the stimulatory effect of Cu on bone regeneration could be associated with its activity on the Wnt/β-catenin axis [172].

The osteogenic, angiogenic, and anti-inflammatory properties of Cu were also highlighted in other recent studies, such as using Cu-doped mineralized diatom (Cu-DBs) [176] and borosilicate glass bone cement [177]. Notably, Li S. and colleagues found that Cu can accelerate bone healing thanks to its anti-inflammatory, angiogenic, and osteogenic properties [177]. Indeed, the controlled release of Cu stimulated the recruitment and polarization of macrophages from the pro-inflammatory phenotype M1 to the anti-inflammatory phenotype M2. Furthermore, the Cu-doped cement showed pro-angiogenic effects by increasing the expressions of angiogenic genes, such as *VEGF* and *fibroblast growth factor 2* (*FGF2*), which encodes the basic fibroblast growth factor (bFGF), in human umbilical cord vein endothelial cells (HUVECs).

Cu role in promoting new bone formation was also investigated through the surface modification of microporous titanium dioxide coating with Cu^2+^ ions [178]. Interestingly, Cu improved the biological activity of titanium alloy since it induced the adhesion, proliferation, and differentiation of MC3T3-E1 cells and promoted the osseointegration of the implant in the rabbit femoral condyle [178]. The potential clinical application of Cu in bone implants was also studied by Wang L. and colleagues. The authors reported that nano-Cu-bearing stainless steel stimulated the enzymatic activity of LOX, promoted the osteogenic differentiation of BMSCs, and accelerated the callus evolution and the healing processes in a rat fracture model [179].

In addition to its beneficial effects on bone, Cu can promote cartilage repair [165]. This makes Cu-based scaffolds potential biomaterials for repairing osteochondral defects, typical of conditions involving both cartilage and subchondral bone, such as OA [165]. As previously mentioned, Cu is a component of LOX, essential for articular cartilage integrity, and plays a key role in the formation of proteoglycan and collagen, which are cartilage matrix constituents [180]. As reported by Makris E. and coworkers, treatment with copper sulfate and hydroxylysine significantly enhanced the enzymatic activity of LOX and collagen cross-links, thus improving the mechanical strength of engineered articular cartilage [181]. In addition to its role in stimulating the synthesis of ECM components, Cu is also involved in cartilage metabolism and promotes chondrocyte proliferation. Previous studies suggested that Cu can stimulate chondrocyte growth and proliferation via enhancing ALP activity and the secretion of IGF-1 and IGF-binding protein 3 (IGFBP-3), which regulate cartilage repair [182,183,184]. Madzovska-Malagurski I. et al. demonstrated that Cu release from alginate microbeads stimulated chondrogenesis in 3D cultures and ECM synthesis [185]. Cu stimulatory effect on chondrogenic differentiation of MSCs was also more recently investigated [186]. As shown by Xu C. and coworkers, Cu can significantly induce chondrogenesis of MSCs, glycosaminoglycan deposition, and the expression of cartilage-related genes (*SOX9*, *ACAN*, and *COL2*) [186]. The beneficial activity of Cu in cartilage lesions was also studied using Cu-incorporated bioactive glass ceramics prepared through a 3D-printing method [187]. Interestingly, Cu accelerated cartilage lesions healing by promoting chondrocytes proliferation and maturation. Moreover, it showed immunomodulatory activity (through HIF signaling pathway activation) and anti-inflammatory properties through the induction of macrophages shifting to the anti-inflammatory phenotype M2, reduction of pro-inflammatory cytokines (such as TNF-α and IL-18), and increasing the expression of the anti-inflammatory cytokine IL-10 [187].

Interestingly, nanoparticles functionalized with copper sulfate and anti-beta-2-microglobulin antibodies (B2M-CuS NPs) showed potential therapeutic efficacy towards OA in a recent in vitro and in vivo study [188]. In particular, B2M-CuS selectively induced apoptosis of senescent chondrocytes (ATDC5 cells) while promoting the chondrogenesis of normal chondrocytes, as observed after intra-articular injection into the knee of OA mice [188]. Another recent study demonstrated that the injection of a thermo-sensitive hydrogel able to release Cu^2+^ ions into the articular cavity of OA rats exerted antioxidant effects by scavenging ROS and RNS species and anti-inflammatory effects by promoting the production of M2 anti-inflammatory macrophages [189]. Table 2 summarizes the main studies performed on Cu-based compounds for bone and cartilage regeneration. Overall, these results showed that Cu can be used to develop novel therapeutic strategies against degenerative joint diseases, such as OA, even though further studies are necessary to determine the exact underlying mechanisms.

## 4. Beneficial Effects of Carnosine and Its Zinc Complex, Polaprezinc, on Bone and Cartilage

**Figure 4:**
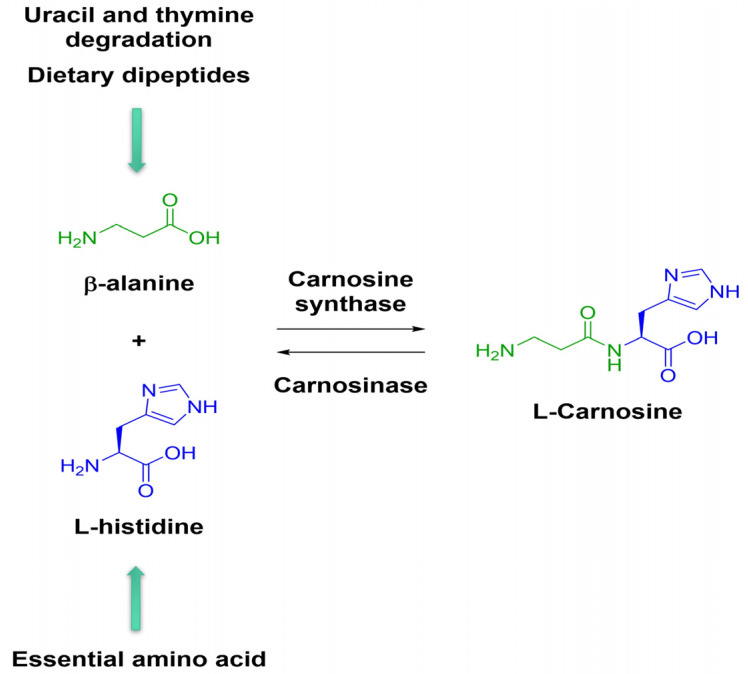
Schematic illustration of carnosine synthesis and degradation. The acquamarine arrows define the type and source of the two amino acids.

Numerous studies show that Car, an endogenous dipeptide and over-the-counter food supplement, and its compounds exert beneficial effects on bone and cartilage under physiological and pathological conditions (Table 3) [9]. Car is present at high levels (in millimolar concentration) in the nervous, cardiac, and muscular tissues [190]. It is synthesized by carnosine synthase 1 (CARNS1) from β-alanine and L-histidine, while its hydrolysis is catalyzed by two isoforms of carnosinases (CN1 and CN2) (Figure 4).

Car is largely studied due to its numerous and always new biological properties [190]. Its pH-buffering activity, the ability to form complexes with metal ions, including Cu^2+^ and Zn^2+^ ions, and its antioxidant, anti-inflammatory, antiaging, antiglycation, and antiaggregating properties are worthy of mention [190]. Furthermore, numerous findings suggest that Car modulates macrophage functions, immune cells activated under inflammatory states, and oxidative stress. RAW 264.7 macrophage cells exposed to lipopolysaccharide and IFN-γ increase Car uptake as an antioxidant defense under pro-inflammatory conditions [191]. Notably, Car demonstrates beneficial effects in the prevention and treatment of pathological conditions, such as neurodegenerative diseases, diabetes, heart failure, circulatory disorders, and cancer [192]. Several studies suggest that Car can also stimulate bone formation by inducing osteoblasts’ proliferation and activity, inhibiting osteoclasts’ bone resorption, and regulating the differentiation of bone marrow MSCs [9]. For instance, Car can promote the differentiation of human periodontal ligament stem cells (PDLSCs) into osteoblasts or chondroblasts and the overall periodontal regeneration. Indeed, Ito-Kato E. and colleagues report that Car upregulates *Runx2/Cbfa1* and *SOX9* mRNA levels and BMP-2 and BMP-7 expression in PDLSCs [193]. As emerged by recent studies, Car could exert beneficial effects in bone disease prevention and treatment thanks to its antioxidant activity, since no specific receptors able to bind Car have been identified so far. In particular, Car can exert antioxidant activity through direct or indirect mechanisms [194]. Indeed, it can directly act as a ROS scavenger thanks to its imidazole ring [195]. Alternatively, it can modulate different molecular pathways, such as Nrf-2 nuclear translocation, which regulates the expressions of antioxidant proteins [19,196]. For instance, Yang Y. et al. investigated the protective effects of Car in a model of OA [197]. Of note is that Car reduces ROS levels, MMP-3 and MMP-13 expression, and NF-KB translocation on IL-1β-induced FLSs, which mediate cartilage destruction and play a crucial role in the pathogenesis of inflammatory diseases, including OA. Oral administration of Car (0.3 and 0.9 g/kg/day for 8 weeks) attenuates the inflammatory response and exerts chondroprotective effects, thus preventing type 2 diabetes-induced OA in rats [197]. Other in vitro and in vivo studies confirm the protective role of Car in OA models. For instance, a recent study suggests that Car alleviates knee OA in FLSs isolated from knee joints and in rats [13]. In particular, Car reduces proinflammatory cytokines and cartilage destruction and protects the synovial membrane by activating the antioxidant Nrf-2/HO-1 axis [13]. In addition, pain-related behavioral experiments show Car’s ability to alleviate the pain associated with knee OA. The therapeutic potential of Car was also investigated in a primary cell culture of chondrocytes and in a model of adjuvant arthritis in rats [198]. Interestingly, Car decreases intracellular oxidant levels in a culture of chondrocytes exposed to hydrogen peroxide, which is involved in RA pathogenesis, suggesting that Car may prevent oxidative injury and tissue damage in RA. In particular, Car displays the maximum antioxidant effect at a concentration of 10 μM. Moreover, Car prevents proteins and lipids oxidative damage and reduces the plasmatic levels of IL-1α, an inflammation marker increased in arthritic animals, after treatment at a concentration of 150 mg/kg/day for 28 days via gastric gavages [198].

Car was also identified as a biomarker of muscle alterations in arthritic mice urine [12]. Metabolomics is an emerging analytical technique used to qualitatively and quantitatively determine small molecule metabolites in biological samples [199]. It represents a novel tool to unveil the relationship between diseased states and metabolic alterations [200]. Changes in the body composition and metabolic profile of RA patients have been investigated to gain insights into the mechanisms involved in the disease’s pathogenesis and to identify potential diagnostic and prognostic markers [201]. Multiple metabolic pathways are altered in RA patients, such as glycolysis, the tricarboxylic acid cycle, the pentose phosphate and arachidonic acid pathways, and amino acid metabolism, leading to abnormal levels of intermediate metabolites. For instance, the metabolic products of tricarboxylic acids, free fatty acids, polyunsaturated fatty acids, prostaglandins, thromboxanes, leukotrienes, and bile acids are increased in RA samples (serum, urine, and synovial fluid) compared to the healthy controls [202]. These metabolic abnormalities can sustain the release of pro-inflammatory mediators, immune cell infiltration, and overall joint disruption [203]. Alabarse P.V.G. et al. analyzed the metabolites in mice urine samples as biomarkers of skeletal muscle loss. To this extent, the authors used collagen-induced arthritis (CIA), a mice model of RA, and control groups. In this study, Car was identified as one of the major metabolites involved in the muscle loss process [12].

Polaprezinc, the Zn complex of Car, has been recently proposed as a drug repurposing candidate for bone fracture healing [17]. It has been used as an orally administered anti-ulcer drug for over 20 years in Japan, where it was first approved in 1994 [204]. Moreover, polaprezinc is used in clinical practice for Zn supplementation in case of Zn deficiency, although it is not approved worldwide [205]. Polaprezinc is also studied for its protective effects on gastrointestinal mucosal and its antioxidant, antiapoptotic, and anti-inflammatory activities [206]. Thanks to the well-known beneficial properties of its constituents, the data in the literature suggest that polaprezinc could be a potential drug candidate for the prevention and treatment of bone diseases characterized by poor osteogenesis and high levels of ROS. Several studies investigated the effects of the Zn-chelating Car on bone tissue and cells. Firstly, the positive effects of polaprezinc on bone metabolism are related to the fact that polaprezinc is easily absorbed in the gastrointestinal tract and enhances Zn bioavailability and uptake in bone tissues [207]. As previously discussed, Zn is a trace mineral element with a fundamental role in skeletal growth, bone homeostasis, and regeneration [208]. Secondly, in vitro studies on MC3T3-E1 cells show that polaprezinc can stimulate osteoblastic differentiation at 10^−7^–10^−5^ M, increasing ALP activity and protein synthesis [209]. This effect is more pronounced for polaprezinc than zinc sulfate at the same concentrations, suggesting a Zn-independent mechanism of action. Moreover, similar to zinc sulfate, polaprezinc significantly increases *Runx2* and *regucalcin* mRNA expression in osteoblastic MC3T3-E1 cells [125]. In addition, the Zn-chelating dipeptide stimulates the differentiation of human periodontal ligament (HPDL) cells into osteoblasts, as previously discussed for Car, via BMP-2 or BMP-7 [210]. Polaprezinc effects on mesenchymal cell differentiation were also studied by Takada T. et al. [211]. Indeed, polaprezinc stimulates C2C12 cell differentiation to osteoblast and chondroblast lineage by increasing *Cbfa1/Runx2*, *ALP*, *SOX9,* and *type X collagen* mRNA expression. Moreover, an in vivo study in weanling rats compared the effects of polaprezinc with zinc sulfate and other zinc-related compounds, such as di(histidino)-zinc and di(N-acetyl-L-alanyl-L-histidinato)-zinc. Polaprezinc administration induces a superior increase in Zn, DNA, calcium contents, and ALP activity compared to the other zinc-based compounds at the same doses [212]. In another study, Kisi S. and Yamaguchi M. show that polaprezinc significantly increased ALP activity in the femoral diaphysis of elderly rats [213].

On the other hand, polaprezinc exerts an inhibitory action on bone resorption. In particular, it inhibits the early stages of osteoclastogenesis by preventing the differentiation of bone marrow cells isolated from rats into osteoclast precursors and blocking the stimulatory effects of PTH and TGF-β on osteoclast-like cell formation [214,215,216,217]. Recently, Ko E.A. and colleagues investigated the influence of polaprezinc on the differentiation of hBMSCs and mouse bone marrow-derived monocytes (mBMMs) [17]. In the first case, polaprezinc upregulates osteogenesis-related genes, such as *Runx2*, *ALP*, *COL1A1*, *SPP1*, *integrin binding sialoprotein (IBSP)*, and *BGLAP*, inducing hBMSCs differentiation into osteoblasts. Interestingly, treatment with the precursor Car does not induce osteogenesis in hBMSCs. Unlike previous studies, Ko E.A. et al. observed that polaprezinc promotes osteoclast differentiation in mBMMs by increasing *NFATc1*, *Cat K*, and *Dcstamp* mRNA expression levels and the transcriptional activity of yes-associated protein (YAP). Although the latter observation contradicts previous studies, the different results may depend on the diverse experimental conditions. Different from polaprezinc, Car treatment does not stimulate osteoclast differentiation, suggesting that polaprezinc effects are probably related to the increased absorption of the metal ion. Moreover, the authors compared polaprezinc effects with those exerted by zinc sulfate. Polaprezinc increases osteogenesis to a greater extent compared to zinc sulfate and exerts different effects on osteoclast differentiation. Indeed, zinc sulfate downregulates NFATc1 and reduces osteoclast activity. In addition, the authors found that oral administration of polaprezinc (25 mg/kg) accelerates healing processes in mice with femoral fractures, as revealed by microCT and histology assays [17]. Overall, this study suggests that polaprezinc could be a good candidate for drug repositioning in patients with bone fractures.

In order to evaluate the potential application of polaprezinc in the field of bone regeneration, the compound was successfully encapsulated into hybrid nanofiber membranes of polycaprolactone/gelatin (PG/0.2–0.8%PZ), and its local anti-osteoporosis and osteoinductive effects were evaluated in vitro and in vivo [218]. Notably, Gao X. et al. showed that the release of polaprezinc exerts antioxidant effects in MC3T3-E1 cells under oxidative conditions via activating the Nrf-2/HO-1/SOD1 pathway and stimulating osteogenic differentiation and proliferation. In particular, the best results were obtained with a loading percentage of polaprezinc equal to 0.4%. Moreover, the polaprezinc-loaded membranes accelerate the repair of cranial bone defects in osteoporotic rats, showing significant clinical potential in oxidative stress-related bone diseases [218].

**Table 3:** Summary of Car and polaprezinc effects on bone and cartilage.

Compound	Experimental Model	Effects	Outcomes	Ref
Car (10^−5^–10^−4^ M)	PDLSCs	↑ *Runx2/Cbfa1* and *SOX9* mRNA ↑ BMP-2 and BMP-7 expression	↑ osteoblast differentiation	[193]
Car (10^−4^ M, 0.1/0.3/0.9 g/kg/day for 8 weeks)	FLSs, diabetes-induced OA in rats	↓ ROS, MMP-3 and MMP-13 expression ↓ NF-KB translocation	chondroprotective effects	[197]
Car (10^−4^ M, 0.5/1.0 g/kg/day for 12 weeks)	FLSs, OA model in rats	↑ Nrf-2/HO-1 signaling pathway ↓ MMP-3 and MMP-13 mRNA expression	protection towards the synovium, ↓ pain	[13]
Car (10^−5^ M, 150 mg/kg for 28 days)	rat primary chondrocytes, rat adjuvant arthritis, model of carrageenan induced hind paw edema	↓ IL-1α, markers of oxidative stress, intracellular oxidant levels	anti-inflammatory, antioxidant activity	[198]
Polaprezinc (10^−7^–10^−5^ M)	MC3T3-E1	↑ ALP activity and protein synthesis	↑ osteoblast differentiation	[209]
Polaprezinc (10^−5^ M)	MC3T3-E1	↑ *Runx2* and *regucalcin* mRNA expression	↑ osteoblast differentiation	[125]
Polaprezinc (10^−5^–10^−4^ M)	HPDL	↑ *Runx2/Cbfa1* and *SOX9*, BMP-2, BMP-7 expression	↑ osteoblast differentiation	[210]
Polaprezinc (10^−5^–10^−4^ M)	C2C12	↑ *Cbfa1/Runx2*, *ALP*, *SOX9* and *type X collagen* mRNA expression	↑ osteoblast and chondrocyte differentiation	[211]
Polaprezinc (2.75 mg Zn/kg/day)	weanling rats	↑ Zn, DNA, calcium contents, and ALP activity	↑ bone metabolism	[212]
Polaprezinc (10^−5^ M)	femoral diaphysis from rats	↑ ALP activity	↑ bone metabolism	[213]
Polaprezinc (10^−7^–10^−5^ M)	mouse marrow cells	↓ osteoclast-like cell formation	↓ bone resorption	[214]
Polaprezinc (10^−6^ M)	mouse marrow cells	↓ PTH stimulatory effects	↓ PTH-stimulated osteoclastogenesis	[215]
Polaprezinc (10^−7^–10^−5^ M)	mouse marrow cells	↓ TGF-β stimulatory effects	↓ osteoclastogenesis	[216]
Polaprezinc (5 × 10^−5^ M)	hBMSCs	↑ ALP activity, ↑ *Runx2*, *ALP*, *COL1A1*, *SPP1*, *IBSP*, *BGLAP* mRNA levels	↑ osteogenic differentiation	[17]
Polaprezinc (5 × 10^−5^ M)	mBMMs	↑ YAP activity, ↑ *NFATc1*, *Cat K*, *Dcstamp* mRNA levels	↑ osteoclastogenic differentiation	[17]
Polaprezinc (25 mg/kg)	mouse femoral fracture model	↑ active bone homeostasis	↑ bone remodeling	[17]
Polaprezinc (PG/0.2–0.8%PZ)	MC3T3-E1, cranial bone defects of osteoporotic rats	↑ Nrf-2/HO-1/SOD1 pathway ↑ ALP activity ↑ osteogenesis	antioxidant effects ↑ osteogenesis ↑ bone regeneration	[218]

Abbreviations: ALP—alkaline phosphatase; BGLAP—bone gamma-carboxyglutamate protein; BMP-2—bone morphogenetic protein 2; BMP-7—bone morphogenetic protein 7; Cat K—cathepsin K; Cbfa1—core binding factor α1; COL1A1—collagen type I alpha 1 chain; Dcstamp—dendrocyte expressed seven transmembrane protein; FLSs—fibroblast-like synoviocytes; hBMSCs—human bone marrow mesenchymal stem cells; HO-1—heme oxygenase-1; HPDL—human periodontal ligament; IBSP—integrin binding sialoprotein; IL-1α—interleukin-1 α; mBMMs—mouse bone marrow-derived monocyte cells; MMP-3—matrix metalloproteinase-3; MMP-13—matrix metalloproteinase-13; NFATc1—Ca^2+^–calcineurin–nuclear factor-activated T cells c1; NF-KB—nuclear factor kappa-light-chain-enhancer of activated B cells; Nrf-2—nuclear factor erythroid 2-related factor; OA—osteoarthritis; PDLSCs—periodontal ligament stem cells; PTH—parathyroid hormone; ROS—reactive oxygen species; Runx2—runt-related transcription factor 2; SOD1—superoxide dismutase 1; SOX9—SRY-box transcription factor 9; SPP1—secreted phosphoprotein 1; TGF-β—transforming growth factor beta; YAP—yes-associated protein. The symbols “↑” and “↓” stand for increase and reduction, respectively.

## 5. Hypothesis on a Potential Exchange of Zinc for Copper by Polarezinc

Car offers five potential metal-binding sites, which are two imidazole nitrogen atoms, a carboxylate group, an amide bond, and a free amino group. Therefore, it acts as a polydentate ligand, forming complexes with tetrahedral or octahedral geometries [219]. Its interactions and chelating properties towards bivalent metal cations, including Cu^2+^ and Zn^2+^, were evaluated and compared. Recently, copper(II) and zinc(II) complexes with Car were characterized using diverse analytical techniques, as reported by Abate C. et al. [220]. In particular, the speciation model and thermodynamic parameters (logβ, Δ*H*, Δ*G*, *T*Δ*S*) of the copper(II) and zinc(II) complex species with Car were determined. Moreover, the sequestering abilities of Car towards Cu^2+^ and Zn^2+^ (pH = 7.4, T = 310.15 K and I = 0.15 mol/L) were compared by calculating the pL_0.5_ (the cologarithm of the ligand concentration able to bind 50% of the metal ion) under physiological-mimicking conditions [220]. The obtained pL_0.5_ values of Car towards Cu^2+^ (7.88) and Zn^2+^ (3.26) show that the dipeptide’s sequestering ability towards Cu^2+^ is approximately five orders of magnitude higher than Zn^2+^.

Cell cultures require optimized media compositions and different reagents, serums, and supplements to proliferate. An aspect often underestimated is the presence of trace metal ions in cell culture media. Although metal ions are necessary for cell viability, they can influence the experimental results. Trace metals were detected in different types of samples using elemental analysis techniques. Studies on trace element content in cell culture media in vitro show variations in Cu, Zn, and other metal ions levels even between batches of the same type of medium from the same supplier [221]. Therefore, metal ion concentrations reported in the catalogs or literature data do not provide an accurate estimation since the baseline levels may vary. In general, the most common growth media used for cell cultures contain Cu at concentrations ranging from 0.04 to 5.20 μM, as reported by Falcone E. et al. [222]. For instance, data from the literature show that minimum essential medium (MEM) and Dulbecco’s modified eagle’s medium (DMEM) contain up to 0.5–0.6 μM of Cu and 0.5–1.74 μM of Zn, respectively [221]. Polaprezinc and Car effects on bone and cartilage were investigated in diverse cell lines, such as MC3T3-E1, hBMSCs, mBMMs, FLSs, and different media, including α-MEM and DMEM (Table 4). The tested concentrations of polaprezinc used in the in vitro studies previously described were in the range 10^−4^–10^−7^ M. Table 4 summarizes the detailed experimental conditions used in in vitro studies that focused on Car and polaprezinc functions in bone and cartilage tissues. Based on the average Cu and Zn content in the cell culture media and the highest sequestering ability of Car towards Cu^2+^ ions, we hypothesize that the beneficial roles exerted by polaprezinc may also be due to a transmetallation process through which Car exchanges Zn^2+^ with Cu^2+^ ions. Indeed, it would be necessary a concentration of Zn five orders of magnitude higher to overcome the greater sequestering ability of Car towards Cu. In addition to the thermodynamic aspect, this hypothesis is further supported by the Irving–Williams stability series (Mn^2+^ < Fe^2+^ < Co^2+^ < Ni^2+^ << Cu^2+^> Zn^2+^), showing that Cu forms complexes with higher stability compared to Zn [223]. In addition, several studies assess that zinc(II) complexes transmetallate with Cu^2+^ ions [224,225,226,227,228]. Moreover, Cu^2+^ ions exert similar effects on bone and cartilage compared to Zn^2+^, as shown in Table 1 and Table 2. Therefore, future studies should investigate whether the beneficial effects of polaprezinc could be related to a transmetallation process. For instance, this hypothesis could be tested by using an extracellular chelator of Cu in vitro that should reduce polaprezinc activity.

## 6. Conclusions

d-block metal ion imbalance occurs in many pathological conditions, including arthritic diseases. Several studies show that an increased Cu/Zn ratio is generally found in the serum of RA subjects, highlighting that these elements could be of value in the diagnosis, prognosis, and evaluation of treatment options for arthritis patients. However, whether altered levels of metal ions are triggering factors or a consequence of the persistent inflammation typical of arthritis is still a matter of debate. Zn and Cu exerts essential modulatory functions on bone and cartilage regeneration, as summarized in Table 1 and Table 2. Car also plays an important role in promoting osteochondral defect healing. Indeed, the dipeptide exerts antioxidant and anti-inflammatory activities in arthritic joints and can help restore cellular metallostasis thanks to its ionophore activity. In addition to its potential therapeutic effects, it could be exploited as a metabolic biomarker in the study of arthritis activity. Notably, its Zn-complex, polaprezinc, bears potential activity in stimulating bone fracture healing. However, Car shows a greater sequestering ability towards Cu compared to Zn, which suggests a potential involvement of Cu in polaprezinc activity in in vitro studies. Indeed, Cu is generally present in cell culture media at concentrations able to displace Zn. As for in vivo experiments, a larger number of factors may be involved compared to in vitro conditions, making difficult the estimation of Cu available to the transmetallation process. Future studies are necessary to verify this hypothesis and gain a more precise interpretation of Cu and Zn levels in RA patients, taking into account not only the metal ions’ concentration in the serum, but also in other tissues.

## Figures and Tables

**Table 2:** Summary of the main properties of Cu-based compounds/materials in bone and cartilage tissues.

Cu Source	Cell Type	Animal Model	Effects	Outcomes	Ref
Cu-titanium implants	MSCs	-	↑ ALP activity, *COL1*, *OPG*, *OPN* expression, ↓ *E. coli* and *S. aureus* growth	↑ osteoblasts proliferation, differentiation, antimicrobial activity	[169]
Cu-titanium implants	BMSCs	rat femur fracture model	↑ Wnt/β-catenin pathway	↑ osteoporotic fractures healing	[172]
Cu-mineralized diatom	PDLSCs	rat cranial defect/full-thickness skin incision models	↑ *ALP*, *Runx2*, *BSP*, *OCN*, *OPN* expression	↑ osteogenesis, angiogenesis	[176]
Cu-borosilicate glass bone cement	HUVECs, hBMSCs	femoral condylar bone defects in rats and rabbits	↑ *Runx2*, *BGLAP*, *SPP1*, *IL-1Ra*, *TGF-β1, VEGF*, *FGF2* ↓ *IL-1β, IL-6* expression	antiinflammatory, angiogenic, osteogenic properties	[177]
Cu- coatings on titanium	MC3T3-E1	rabbit femoral condyle	↑ cell adhesion, proliferation, differentiation	↑ bone formation ↑ osseointegration	[178]
Nano Cu- bearing stainless steel	BMSCs	rat fracture model	↑ LOX activity, osteogenic differentiation ↑ callus evolution	↑ bone formation, fracture healing	[179]
Copper sulfate	chondrocytes from bovine joints	-	↑ LOX activity	↑ biomechanical properties in engineered neocartilage	[181]
Copper sulfate	chondrocytes from piglets	-	↑ ALP activity, collagen content	↑ chondrocytes proliferation	[182]
Copper sulfate	chondrocytes from pigs	-	↑ IGF-1 and IGFBP-3 secretion	↑ chondrocytes proliferation	[183]
Cu-alginate hydrogels	bovine calf chondrocytes	-	↑ cell proliferation, ECM synthesis	↑ chondrogenesis	[185]
Cu-alginate porous scaffold	MSCs	mice muscle pouch	↑ extracellular GAG deposition, ↑ *SOX9*, *ACAN*, *COL2* expression	↑ chondrogenic differentiation, ↑ chondrogenesis	[186]
Cu-bioactive glass ceramics	rabbit chondrocytes, RAW264.7 cells	rabbit osteochondral defect model	↑ HIF pathway ↑ anti-inflammatory cytokines	↑ osteochondral regeneration, ↓ inflammation	[187]
Copper sulfide nanoparticles	ATDC5	OA mouse model	↑ GAG and COL2 synthesis, ↑ apoptosis in senescent chondrocytes	↑ cartilage regeneration	[188]
Cu-doped hydrogel	chondrocytes, L929, RAW264.7	rat OA model	ROS scavenging, macrophages repolarization	antioxidant activity, ↓ cartilage degradation, ↓ inflammation	[189]

Abbreviations: ACAN—aggrecan; ALP—alkaline phosphatase; BGLAP—bone gamma-carboxyglutamate protein; BMSCs—bone marrow stromal cells; BSP—bone sialoprotein; COL1—collagen type 1; COL2—collagen type 2; ECM—extracellular matrix; FGF2—fibroblast growth factor 2; GAG—glycosaminoglycan; hBMSCs—human bone marrow mesenchymal stem cells; HIF—hypoxia-inducible factors; HUVECs—human umbilical cord vein endothelial cells; IGF-1—insulin-like growth factor-1; IGFBP-3—IGF-binding protein 3; IL-1Ra—interleukin-1 receptor antagonist; IL-1β—interleukin-1 beta; IL-6—interleukin 6; LOX—lysyl oxidase; MSCs—mesenchymal stem/stromal cells; OA—osteoarthritis; OCN—osteocalcin; OPG—osteoprotegerin; OPN—osteopontin; PDLSCs—periodontal ligament stem cells; ROS—reactive oxygen species; Runx2—runt-related transcription factor 2; SOX9—SRY-box transcription factor 9; SPP1—secreted phosphoprotein 1; TGF-β1—transforming growth factor beta 1; VEGF—vascular endothelial growth factor; Wnt—wingless-related integration site. The symbols “↑” and “↓” stand for increase and reduction, respectively.

**Table 4:** Experimental conditions of in vitro studies investigating polaprezinc and Car effects on bone and cartilage.

Compound (Tested Conc.)	Cell Type	Medium	Serum	Supplements	Duration	Ref.
Polaprezinc (5 × 10^−5^ M)	hBMSCs	DMEM-LG	10% FBS	1% antibiotic– antimycotic solution, 10 mM β-glycerophosphate, 50 μg/mL ascorbic acid	5–12 days	[17]
Car (5 × 10^−5^ M)	hBMSCs	DMEM-LG	10% FBS	1% antibiotic– antimycotic solution, 10 mM β-glycerophosphate, 50 μg/mL ascorbic acid	5–12 days	[17]
Polaprezinc (5 × 10^−5^ M)	mBMMs	α-MEM	10% FBS	1% antibiotic– antimycotic solution, 10 ng/mL mM-CSF and 10 ng/mL mRANKL	-	[17]
Car (5 × 10^−5^ M)	mBMMs	α-MEM	10% FBS	1% antibiotic–antimycotic solution, 10 ng/mL mM-CSF and 10 ng/mL mRANKL	-	[17]
Car (10^−4^–10^−5^ M)	PDLSCs	α-MEM	10% FBS	antibiotics	up to 10 days	[193]
Car (10^−4^ M)	FLSs	DMEM	10% FBS	antibiotics (100 U/mL penicillin and 100 mg/mL streptomycin)	-	[197]
Car (10^−4^ M)	FLSs	DMEM	-	antibiotics penicillin and streptomycin (100 mg/mL)	-	[13]
Car (10^−5^ M)	Rat primary chondrocytes	DMEM/ Ham’s F-12	10% FBS	1 mmol/L glutamine, 100 U/mL penicillin, 100 μg/mL streptomycin	-	[198]
Polaprezinc (10^−7^–10^−5^ M)	MC3T3-E1	α-MEM	10% FBS	-	up to 10 days	[209]
Polaprezinc (10^−4^–10^−5^ M)	PDLSCs	α-MEM	10% FBS	1% (vol/vol) penicillin streptomycin solution (5000 units/mL penicillin and 50 mg/mL streptomycin)	up to 10 days	[210]
Polaprezinc (10^−7^–10^−5^ M)	Rat marrow culture	α-MEM	10% HI FBS	-	7 days	[214]
Polaprezinc (10^−7^–10^−5^ M)	osteoclasts isolated from rat femoral tissues	α-MEM	10% FBS	-	24 h	[214]
Polaprezinc (10^−6^ M)	Bone marrow cells isolated from mice	α-MEM	10% HI FBS	Penicillin–streptomycin solution (5000 U/mL penicillin; 5000 pg/mL streptomycin)	7 days	[215]
Polaprezinc (10^−6^ M)	Bone marrow cells isolated from mice	α-MEM	10% HI FBS	Penicillin–streptomycin solution (5000 U/mL penicillin; 5000 pg/mL streptomycin)	7 days	[216]
Polaprezinc (10^−5^ M)	MC3T3-E1	α-MEM	10% FBS	Penicillin–streptomycin (5000 U/mL penicillin; 5000 lg/mL streptomycin)	24–72 h	[125]

Abbreviations: α-MEM—α minimum essential medium; DMEM—Dulbecco’s modified eagle’s medium; FBS—fetal bovine serum; FLSs—fibroblast-like synoviocytes; hBMSCs—human bone marrow mesenchymal stem cells; HI—heat-inactivated; LG—low glucose; mBMMs—mouse bone marrow-derived monocyte cells; mM-CSF—mouse macrophage-colony stimulating factor; PDLSCs—periodontal ligament stem cells; RANKL—receptor activator of NF-KB ligand.

## Data Availability

Not applicable.
